# Supplementary material for: Young Adults Rehabilitation experiences and Needs following Stroke (YARNS): A scoping review of the rehabilitation care experiences and outcomes of young adults post-stroke
Source: PLoS One. 2025 Jan 31;20(1):e0279523. doi: 10.1371/journal.pone.0279523 (PMC11785345; doi:10.1371/journal.pone.0279523)
Supplement: S1 Table — (PDF) [file pone.0279523.s001.pdf]

S1 Table: Research questions, operational definitions and search terms

Research questions	Operational definitions	Search terms
What is the impact of stroke on young adults?	Impact: any consequences caused by stroke.<br>Definition of stroke is based on the American Heart Association/American Stroke Association [119]:<br>- Central nervous system (CNS) infarction<br>- Ischemic stroke<br>- Silent CNS infarction<br>- Intracerebral haemorrhage<br>- Silent cerebral haemorrhage<br>- Subarachnoid haemorrhage<br>- Cerebral venous thrombosis	‘stroke’, ‘cerebrovascular accident’, ‘brain injury’, ‘thrombosis’, ‘infarction’, ‘acute cerebrovascular events’, ‘apoplexy’, ‘CVA’, ‘cerebral stroke’, ‘brain vascular accident’, ‘cerebrovascular disorders’, ‘covert brain injury’<br>‘isch(a)emic’<br>‘intracerebral’<br>‘central nervous system infarction’<br>‘subarachnoid’<br>‘h(a)morrhage’<br>‘Intraparenchymal’<br>‘silent cerebral’<br>‘cerebral venous’
What are the focus and the expected outcomes of stroke rehabilitation in young adults?	Definition of stroke rehabilitation based on NICE [120] guideline for stroke rehabilitation in adults: *Stroke rehabilitation is a multidimensional process, which is designed to facilitate restoration of, or adaptation to the loss of, physiological or psychological function when reversal of the underlying pathological process is incomplete. Rehabilitation aims to enhance functional activities and participation in society and thus improve quality of life*.	‘Occupational therapy’, ‘stroke rehabilitation’, ‘post stroke’, ‘rehabilitation’, ‘recovery’
	Outcomes: Any reported outcome measures following stroke including but not limited to functional and disability outcomes and quality of life	‘functional’, ‘outcome’, ‘disability’, ‘outcome measure', ‘short’, ‘long’, ‘short-term’, ‘short term’, ‘long-term’, ‘long term’

Research questions	Operational definitions	Search terms
What are young adults' experiences of stroke rehabilitation care in acute and health and social care settings?	Acute care settings:<br>- Inpatient<br>- Inpatient rehabilitation	'hospital', 'inpatient'
	Social care settings:<br>- Outpatient rehabilitation<br>- Community-based<br>- Nursing home<br>- Primary care	'community', 'community-based'<br>'outpatient', 'clinic'<br>'social care'

Abbreviations: CNS = Central Nervous System, CVA = Cerebrovascular Accident
