# Supplementary material for: Young Adults Rehabilitation experiences and Needs following Stroke (YARNS): A scoping review of the rehabilitation care experiences and outcomes of young adults post-stroke
Source: PLoS One. 2025 Jan 31;20(1):e0279523. doi: 10.1371/journal.pone.0279523 (PMC11785345; doi:10.1371/journal.pone.0279523)
Supplement: S2 Table — (PDF) [file pone.0279523.s002.pdf]

S2 Table: Search Strategy

CINAHL

S1 (MH "Cerebrovascular Disorders+")

S2 stroke* OR "cerebrovascular accident*" OR CVA OR "cerebral vascular event*" OR CVE OR "cerebrovascular event*" OR apoplexy OR "cerebrovascular injur*"

S3 (brain OR cerebr* OR head OR subarachnoid OR Intraparenchymal) N3 (haemorrhage* OR hemorrhage* OR ischemic OR ischaemic OR infarction* OR thrombosis OR thrombotic OR accident* OR disorder* OR injur*)

S4 S1 OR S2 OR S3

S5 rehabilitation OR therap* OR recovery OR "post stroke" OR "stroke survivor*" OR physiotherap* OR "following stroke" OR "after stroke”

S6 experience* or attitude* or perception* or perceive* or belief*

S7 hospital* OR "acute care" OR inpatient* OR "stroke unit*" OR "stroke cent*" OR ward OR "care facilit*" OR "health facilit*

S8 communit* OR "community based" OR population OR "population based"

S9 S7 OR S8

S10 S4 AND S5 AND S6

S11 S9 AND S10

S12 S9 AND S10

S13 "traumatic brain injur*" OR TBI or "head injur*" OR concussion OR "cerebral palsy"

S14 s12 NOT S13

S15 S14 NOT ("child*" or "adolesce*" or "teenager*" OR "pediatr*" or "elder*" or "aged patient*")

S16 S15 NOT ("cardiac arrest" or "heart arrest" or "cardiopulmonary arrest" OR "myocard* infarction" OR MCI)

S17 S16 NOT ("postoperative" or "post operat*" or "post surg*")

S18 S17 NOT (oncology OR carcinoma OR cancer OR "radio therap*" OR "schizophrenia" OR "brain tumor")

Limiters – Publication Year: 2000-2022

OVID, including AMED, MEDLINE, EMBASE, PsycINFO

Search Strategy:

1 (hospital* or "acute care" or inpatient* or "stroke unit*").mp. [mp=ab, hw, ti, tc, id, ot, tm, mh, tn, dm, mf, dv, kw, fx, dq, nm, kf, ox, px, rx, ui, sy]

2 exp Stroke/

3 (rehabilitation or therap* or recovery or "post stroke" or "stroke survivor*" or physiotherap*).mp. [mp=ab, hw, ti, tc, id, ot, tm, mh, tn, dm, mf, dv, kw, fx, dq, nm, kf, ox, px, rx, ui, sy]

4 stroke*.mp. [mp=ab, hw, ti, tc, id, ot, tm, mh, tn, dm, mf, dv, kw, fx, dq, nm, kf, ox, px, rx, ui, sy]

5 2 or 4

6 1 and 3 and 5

7 ("post stroke" or "stroke survivor*" or "stroke rehabilitation" or "stroke recover*" or "following stroke*").mp. [mp=ab, hw, ti, tc, id, ot, tm, mh, tn, dm, mf, dv, kw, fx, dq, nm, kf, ox, px, rx, ui, sy]

8 1 and 7

9 (experience* or attitude* or perception* or perceive* or belief*).mp. [mp=ab, hw, ti, tc, id, ot, tm, mh, tn, dm, mf, dv, kw, fx, dq, nm, kf, ox, px, rx, ui, sy]

10 1 and 7 and 9

11 remove duplicates from 10

12 7 and 9

13 "review*".m_titl.

14 7 and 13

OVID including AMED, MEDLINE, EMBASE, PsycINFO

1. (hospital* or "acute care" or inpatient* or "stroke unit*" or "stroke cent*" or ward*).mp. [mp=ab, hw, ti, tn, ot, dm, mf, dv, kw, fx, dq, nm, kf, ox, px, rx, ui, sy, tc, id, tm, mh]

2. exp Stroke/

3. ("stroke rehabilitation" or "stroke therap*" or "stroke recover*" or "post stroke" or "stroke survivor*" or "stroke physiotherap*" or "following stroke" or "after stroke" or "stroke physical therap*").mp. [mp=ab, hw, ti, tn, ot, dm, mf, dv, kw, fx, dq, nm, kf, ox, px, rx, ui, sy, tc, id, tm, mh]

4. (experience* or attitude* or perception* or perceive* or belief*).mp. [mp=ab, hw, ti, tn, ot, dm, mf, dv, kw, fx, dq, nm, kf, ox, px, rx, ui, sy, tc, id, tm, mh]

5. (communit* or "community based" or population or "population based").mp. [mp=ab, hw, ti, tn, ot, dm, mf, dv, kw, fx, dq, nm, kf, ox, px, rx, ui, sy, tc, id, tm, mh]

6. 1 or 5

7. 2 and 3 and 4

8. 6 and 7

9. limit 8 to english language

10. limit 9 to yr="2000 -Current"

11. remove duplicates from 10

12. ("traumatic brain injur*" or TBI or "head injur*" or concussion or "cerebral palsy").mp. [mp=ab, hw, ti, tn, ot, dm, mf, dv, kw, fx, dq, nm, kf, ox, px, rx, ui, sy, tc, id, tm, mh]

13. 11 not 12

14. 13 not ("child*" or "adolesce*" or "teenager*" or "pediatr*" or "elder*" or "aged patient*").mp. [mp=ab, hw, ti, tn, ot, dm, mf, dv, kw, fx, dq, nm, kf, ox, px, rx, ui, sy, tc, id, tm, mh]

15. 14 not ("cardiac arrest" or "heart arrest" or "cardiopulmonary arrest" or "myocard* infarction" or MCI).mp. [mp=ab, hw, ti, tn, ot, dm, mf, dv, kw, fx, dq, nm, kf, ox, px, rx, ui, sy, tc, id, tm, mh]

16. 15 not ("postoperative" or "post operat*" or "post surg*").mp. [mp=ab, hw, ti, tn, ot, dm, mf, dv, kw, fx, dq, nm, kf, ox, px, rx, ui, sy, tc, id, tm, mh]

17. 16 not (oncology or carcinoma or cancer or "radio therap*" or "schizophrenia" or "brain tumor").mp. [mp=ab, hw, ti, tn, ot, dm, mf, dv, kw, fx, dq, nm, kf, ox, px, rx, ui, sy, tc, id, tm, mh]

ASSIA

(((((communit* OR "community based" OR population OR "population based" OR "social care") AND la.exact("English")) OR ((hospital* OR "acute care" OR inpatient* OR ("stroke unit") OR ("stroke center" OR "stroke centers") OR ward OR ("care facilities" OR "care facility") OR ("health facilities" OR "health facility")) AND la.exact("English"))) AND ((rehabilitation OR therap* OR recovery OR "post stroke" OR ("stroke survivors") OR physiotherap* OR "following stroke" OR "after stroke") AND la.exact("English")) AND ((stroke* OR ("cerebrovascular accident") OR CVA OR "cerebral vascular event*" OR CVE OR "cerebrovascular event*" OR apoplexy OR "cerebrovascular injur*") AND la.exact("English")) AND ((experience* OR attitude* OR perception* OR perceive* OR belief*) AND la.exact("English"))) NOT "traumatic brain injur*") NOT (Child* OR pediatr* OR adolescen* OR elder* OR "older adult*" OR "older patient*" OR "myocardial infarction")

Limited by: Date: From 2000 to 2022; Language: English

x

Cochrane Library

#1 hospital* or "acute care" or inpatient* or "stroke unit*" or "stroke cent*" or ward*

#2 "stroke rehabilitation" or "stroke therap*" or "stroke recover*" or "post stroke" or "stroke survivor*" or "stroke physiotherap*" or "following stroke" or "after stroke" or "stroke physical therap*"

#3 stroke

#4 experience* or attitude* or perception* or perceive* or belief*

#5 #2 AND #3 AND #4

#6 communit* or "community based" or population or "population based"

#7 #1 OR #6

#8 #5 AND #7 with Cochrane Library publication date Between Jan 2000 and April 2022

PEDro

Abstract & Title: Stroke AND adult

Subdiscipline: neurology

Published since: 2000

Web of Science

Indexes=SCI-EXPANDED, SSCI, A&HCI, CPCI-S, CPCI-SSH, BKCI-S, BKCI-SSH, ESCI, CCR-EXPANDED, IC

Timespan=2000-2022

1 TOPIC: (stroke)

2 TOPIC: ("brain injury")

3 TOPIC: ("cerebrovascular accident")

4 TOPIC: ("central nervous system infarction")

5 TOPIC: ("isch(a)emic")

6 TOPIC: ("ischaemic")

7 TOPIC: ("haemorrhage")

8 TOPIC: (hospital)

9 TOPIC: (inpatient)

10 TOPIC: (community)

11 TOPIC: ("community-based")

12 TOPIC: (outpatient)

13 TOPIC: (clinic)

14 TOPIC: ("social care")

15 TOPIC: ("occupational therapy")

16 TOPIC: ("stroke rehabilitation")

17 TOPIC: ("post stroke")

18 TOPIC: (rehabilitation)

19 TOPIC: (recovery)

20 TOPIC: (functional)

21 TOPIC: (outcome)

22 TOPIC: (disability)

23 TOPIC: ("outcome measure")

24 TOPIC: (short-term)

25 TOPIC: ("short term")

26 TOPIC: ("long term")

27 TOPIC: (long-term)

28 #7 OR #6 OR #4 OR #3 OR #2 OR #1

29 #14 OR #13 OR #12 OR #11 OR #10 OR #9 OR #8

30 #19 OR #18 OR #17 OR #16 OR #15

31 #27 OR #26 OR #25 OR #24 OR #23 OR #22 OR #21 OR #20

32 #31 AND #21 AND #20 AND #19 AND #18 AND #17

33 #31 AND #30 AND #29 AND #28

34 #7 OR #6 OR #2 OR #1

35 #19 OR #18

36 #35 AND #34 AND #31 AND #29 AND #21

37 TOPIC: ("young adult")

38 #37 AND #36

39 TOPIC: ("young")

40 TOPIC: (experience)

41 #40 AND #36

42 #41 AND #39
