# Supplementary material for: Young Adults Rehabilitation experiences and Needs following Stroke (YARNS): A scoping review of the rehabilitation care experiences and outcomes of young adults post-stroke
Source: PLoS One. 2025 Jan 31;20(1):e0279523. doi: 10.1371/journal.pone.0279523 (PMC11785345; doi:10.1371/journal.pone.0279523)
Supplement: Attachment — Submitted filename: Response to Reviewers.docx [file pone.0279523.s004.docx]

**Response to reviewers**
(all line numbers refer to the ‘Revised Manuscript with Track Changes’)

The response is structured using each point of the reviewers’ feedback with a response in italics below it.

**Journal Requirements:**

Please review your reference list to ensure that it is complete and correct. If you have cited papers that have been retracted, please include the rationale for doing so in the manuscript text, or remove these references and replace them with relevant current references. Any changes to the reference list should be mentioned in the rebuttal letter that accompanies your revised manuscript. If you need to cite a retracted article, indicate the article’s retracted status in the References list and also include a citation and full reference for the retraction notice.

*Response*

*We have checked all references and replaced two references due to the noted reasons:*

*12.*	*Scottish Intercollegiate Guidelines Network (SIGN). Management of Patients with Stroke: Rehabilitation, Prevention and Management of Complications, and Discharge Planning. 2010.*

*Reason: The guideline was superseded by the National Clinical Guideline for the United Kingdom and Ireland. 2023*

*30.*	*YARNS - Young Adults Rehabilitation experiences and Needs following Stroke [Internet]. The University of Edinburgh. 2020 [cited 2020 Nov 2]. Available from: https://www.ed.ac.uk/health/yarns.*

*Reason: The link has been moved to the following:* **<https://www.ed.ac.uk/health/research/current-research/yarns-transitions/yarns-2020-21>**

*We confirm that the reference list has been updated accordingly. In addition, we also edited typos on lines 113 and 186.*

**Reviewers' comments:**

Reviewer's Responses to Questions

**Comments to the Author**

1. If the authors have adequately addressed your comments raised in a previous round of review and you feel that this manuscript is now acceptable for publication, you may indicate that here to bypass the “Comments to the Author” section, enter your conflict of interest statement in the “Confidential to Editor” section, and submit your "Accept" recommendation.

Reviewer #2: (No Response)

Reviewer #3: All comments have been addressed

2. Is the manuscript technically sound, and do the data support the conclusions?

The manuscript must describe a technically sound piece of scientific research with data that supports the conclusions. Experiments must have been conducted rigorously, with appropriate controls, replication, and sample sizes. The conclusions must be drawn appropriately based on the data presented.

Reviewer #2: Yes

Reviewer #3: Yes

3. Has the statistical analysis been performed appropriately and rigorously?

Reviewer #2: (No Response)

Reviewer #3: Yes

4. Have the authors made all data underlying the findings in their manuscript fully available?

The [PLOS Data policy](https://eur02.safelinks.protection.outlook.com/?url=http%3A%2F%2Fwww.plosone.org%2Fstatic%2Fpolicies.action%23sharing&data=05%7C02%7CColin.Chandler%40ed.ac.uk%7C87d54dcc81404e1f4a2f08dc22821627%7C2e9f06b016694589878910a06934dc61%7C1%7C0%7C638423189309686154%7CUnknown%7CTWFpbGZsb3d8eyJWIjoiMC4wLjAwMDAiLCJQIjoiV2luMzIiLCJBTiI6Ik1haWwiLCJXVCI6Mn0%3D%7C0%7C%7C%7C&sdata=NU1PnkAfHElxqEhP%2BQJGXuYvNE7SkmcjEfh3iLIzmOQ%3D&reserved=0) requires authors to make all data underlying the findings described in their manuscript fully available without restriction, with rare exception (please refer to the Data Availability Statement in the manuscript PDF file). The data should be provided as part of the manuscript or its supporting information, or deposited to a public repository. For example, in addition to summary statistics, the data points behind means, medians and variance measures should be available. If there are restrictions on publicly sharing data—e.g. participant privacy or use of data from a third party—those must be specified.

Reviewer #2: Yes

Reviewer #3: Yes

5. Is the manuscript presented in an intelligible fashion and written in standard English?

PLOS ONE does not copyedit accepted manuscripts, so the language in submitted articles must be clear, correct, and unambiguous. Any typographical or grammatical errors should be corrected at revision, so please note any specific errors here.

Reviewer #2: Yes

Reviewer #3: Yes

6. Review Comments to the Author

Please use the space provided to explain your answers to the questions above. You may also include additional comments for the author, including concerns about dual publication, research ethics, or publication ethics. (Please upload your review as an attachment if it exceeds 20,000 characters)

Reviewer #2: Thank you for submitting an improved manuscript. There are just a few points for consideration:

1. The sentence on lines 41-42 puts positive impact before negative, whereas the greatest weight of impact, and the focus of rehabilitation, is rightly on the negative effects, and this is the focus of the paper.

The Impact section on lines 191 et seq is much more appropriately balanced. Thus the sentence on lines 41-42 needs removing, and the point about some positive impacts put as a clause towards the end of the Abstract's Results section.

*R2-1* *Response:*

*Thank you for this comment. We have now revised the sentences on lines 42-44 as suggested.*

2. Line 181 - the comma is misplaced, and should appear after "literature".

*R2-2 Response:*

*Thank you for catching our typo. We have now revised the comma (line 187 in the corrected manuscript version).*

3. Line 241 - the dash is inappropriate and should be removed.

*R2-3 Response:*
*Thank you again for noticing this mistake. We have deleted the dash. (now line 247)*

4. "wolf" should be "Wolf" (author's name).

*R2-4 Response:*
*Thank you. We have now capitalised ‘Wolf’ (line 331).*

5. I still consider that in the Discussion there should be some recognition that health, care, and social welfare systems are not universally homogenous across the globe, and their nature may have influence on the outcome of service provision in individual countries or settings.

*R2-5 Response:*

*Thank you for the valuable suggestion. We have included a statement acknowledging the diverse healthcare systems in the included articles on lines 637-640.*

Reviewer #3: Thank you for the opportunity to read an interesting paper on Stroke. The paper is written clearly with lots of information to read about.
1. The research question is clear and appropriate for the scoping review.
2. The abstract delivers adequate information for a reader to understand the entire paper.
3. Introduction: Well written and delivers adequate overview of the condition.
4. Methods:

I have only minor comments to address:
The review was structured on Arksey and O'Malley methodological framework for scoping studies. The framework has six stages: The paragraphs that are written under the methodology can be fit under the six stages listed below which makes a reader to better understand the steps that were followed. For example: 1. Identifying the research question—the paragraph can have a heading---similar to follow for the others.

*R3-1 Response:*

*We appreciate this suggestion and have taken it on board by adding sub-headings that reflect the stages of Arksey and O’Malley’s framework. (Lines 87, 97, 108, 124, 150, 157)*

Name of the databases can be elaborated at the first use CINAHL (Cumulative Index to Nursing and Allied Health Literature)

*R3-2 Response:*

*Thank you for the helpful suggestion. We have included the database abbreviations (lines 100-103).*

The authors describe as All types of publications in journal articles---could it be mentioned as original studies or reviews.

*R3-3 Response:*

*We agree with this suggestion and have amended Table 3 (line 195) by replacing 'journal article' with 'original study' accordingly.R3-3 Response:*

5. Results:
Line 184, the authors describe as majority of studies could it be replaced as most of the studies?

*R3-4 Response:*

*Thank you for this comment. We have amended the sentence (189-190) based on your suggestion.*

6. Discussion is well -written and it justifies the purpose of this review.

*R3-5 Response: Thank you for this comment*

7. PLOS authors have the option to publish the peer review history of their article ([what does this mean?](https://eur02.safelinks.protection.outlook.com/?url=https%3A%2F%2Fjournals.plos.org%2Fplosone%2Fs%2Feditorial-and-peer-review-process%23loc-peer-review-history&data=05%7C02%7CColin.Chandler%40ed.ac.uk%7C87d54dcc81404e1f4a2f08dc22821627%7C2e9f06b016694589878910a06934dc61%7C1%7C0%7C638423189309691883%7CUnknown%7CTWFpbGZsb3d8eyJWIjoiMC4wLjAwMDAiLCJQIjoiV2luMzIiLCJBTiI6Ik1haWwiLCJXVCI6Mn0%3D%7C0%7C%7C%7C&sdata=4f74%2FmrHO%2BCX9uf1vbk%2F5rcnhfnjxV62EuH%2Bv56piv0%3D&reserved=0)). If published, this will include your full peer review and any attached files.


If you choose “no”, your identity will remain anonymous but your review may still be made public.


**Do you want your identity to be public for this peer review?** For information about this choice, including consent withdrawal, please see our [Privacy Policy](https://eur02.safelinks.protection.outlook.com/?url=https%3A%2F%2Fwww.plos.org%2Fprivacy-policy&data=05%7C02%7CColin.Chandler%40ed.ac.uk%7C87d54dcc81404e1f4a2f08dc22821627%7C2e9f06b016694589878910a06934dc61%7C1%7C0%7C638423189309697915%7CUnknown%7CTWFpbGZsb3d8eyJWIjoiMC4wLjAwMDAiLCJQIjoiV2luMzIiLCJBTiI6Ik1haWwiLCJXVCI6Mn0%3D%7C0%7C%7C%7C&sdata=eq3NuMNfkhf1DJcSi7CGro7tprTpru9ezpHFwJlQVrg%3D&reserved=0).

Reviewer #2: No

Reviewer #3: No
