# Supplementary material for: Young Adults Rehabilitation experiences and Needs following Stroke (YARNS): A scoping review of the rehabilitation care experiences and outcomes of young adults post-stroke
Source: PLoS One. 2025 Jan 31;20(1):e0279523. doi: 10.1371/journal.pone.0279523 (PMC11785345; doi:10.1371/journal.pone.0279523)
Supplement: Attachment — Submitted filename: Response to reviewers.docx [file pone.0279523.s005.docx]

**Response to reviewers**

The response is structured using each point of the reviewers’ feedback with a response in italics below it.

**Journal Requirements:**

Please review your reference list to ensure that it is complete and correct. If you have cited papers that have been retracted, please include the rationale for doing so in the manuscript text, or remove these references and replace them with relevant current references. Any changes to the reference list should be mentioned in the rebuttal letter that accompanies your revised manuscript. If you need to cite a retracted article, indicate the article’s retracted status in the References list and also include a citation and full reference for the retraction notice.

*Response*

*We have checked all references and updated two for the following reasons:*

*3.	‘Stroke Association. State of the Nation: Stroke Statistics 2018’*

*Reason: The page is no longer available and has been replaced by ‘Stroke Association. Stroke Statistics’ this page is not dated, but appears to be based on the 2018 data. The web link and date accessed have replaced and updated reference 3.*

*120.	National Institute for Health and Care Excellence (NICE). Stroke rehabilitation in adults 2013*

*Reason: The guideline has been updated and is now Nice Guideline [NG236], reference is updated to reflect this.*

*We confirm that the reference list has been updated accordingly.*

**Reviewers' comments:**

Reviewer's Responses to Questions

**Comments to the Author**

1. If the authors have adequately addressed your comments raised in a previous round of review and you feel that this manuscript is now acceptable for publication, you may indicate that here to bypass the “Comments to the Author” section, enter your conflict of interest statement in the “Confidential to Editor” section, and submit your "Accept" recommendation.

Reviewer #2: All comments have been addressed

Reviewer #4: (No Response)

2. Is the manuscript technically sound, and do the data support the conclusions?

The manuscript must describe a technically sound piece of scientific research with data that supports the conclusions. Experiments must have been conducted rigorously, with appropriate controls, replication, and sample sizes. The conclusions must be drawn appropriately based on the data presented.

Reviewer #2: Yes

Reviewer #4: Yes

3. Has the statistical analysis been performed appropriately and rigorously?

Reviewer #2: N/A

Reviewer #4: Yes

4. Have the authors made all data underlying the findings in their manuscript fully available?

The [PLOS Data policy](https://eur02.safelinks.protection.outlook.com/?url=http%3A%2F%2Ftrack.editorialmanager.com%2FCL0%2Fhttp%3A%252F%252Fwww.plosone.org%252Fstatic%252Fpolicies.action%2523sharing%2F1%2F010f01933d937f8d-58e7117c-50b5-4d8a-a0aa-274a8bf2e47d-000000%2FL_RuYdcu8JeDBgU0etcM6dM8WVFcvHS4Y3Y4XP7C49g%3D185&data=05%7C02%7C%7Cef742d632d6d482b2a3108dd0903fc93%7C2e9f06b016694589878910a06934dc61%7C0%7C0%7C638676634923423559%7CUnknown%7CTWFpbGZsb3d8eyJFbXB0eU1hcGkiOnRydWUsIlYiOiIwLjAuMDAwMCIsIlAiOiJXaW4zMiIsIkFOIjoiTWFpbCIsIldUIjoyfQ%3D%3D%7C0%7C%7C%7C&sdata=YdvTpPqKhJSotB4kUmd%2Bzh%2BBXNfhjHr1EmwWGWnKC2k%3D&reserved=0) requires authors to make all data underlying the findings described in their manuscript fully available without restriction, with rare exception (please refer to the Data Availability Statement in the manuscript PDF file). The data should be provided as part of the manuscript or its supporting information, or deposited to a public repository. For example, in addition to summary statistics, the data points behind means, medians and variance measures should be available. If there are restrictions on publicly sharing data—e.g. participant privacy or use of data from a third party—those must be specified.

Reviewer #2: Yes

Reviewer #4: Yes

5. Is the manuscript presented in an intelligible fashion and written in standard English?

PLOS ONE does not copyedit accepted manuscripts, so the language in submitted articles must be clear, correct, and unambiguous. Any typographical or grammatical errors should be corrected at revision, so please note any specific errors here.

Reviewer #2: Yes

Reviewer #4: Yes

6. Review Comments to the Author

Please use the space provided to explain your answers to the questions above. You may also include additional comments for the author, including concerns about dual publication, research ethics, or publication ethics. (Please upload your review as an attachment if it exceeds 20,000 characters)

Reviewer #2: Thank you for fully implementing the previous feedback. This thoroughness is appreciated and improves a good manuscript.

Reviewer #4: Overview
First, I commend the authors for the efforts and rigour invested into this study. This scoping review sought to explore young adults rehabilitation experiences and needs following stroke (YARNS): A scoping review of the rehabilitation care experiences and outcomes of young adults post-stroke. According to the authors,” results highlight the unmet needs of young adults in their stroke recovery journey”. Authors concluded thus “Effective rehabilitation programmes and interventions should be developed to support young adults following stroke and meet their age-specific needs”

Areas of improvements
This is elegant piece of literature. The methodology was sound and the theoretical framework applied throughout the study. Also, the language is standard. However, a few minor concerns have been highlighted in the manuscript text.

Decision
I recommend the publication of the manuscript following minor revision.

7. PLOS authors have the option to publish the peer review history of their article ([what does this mean?](https://eur02.safelinks.protection.outlook.com/?url=https%3A%2F%2Ftrack.editorialmanager.com%2FCL0%2Fhttps%3A%252F%252Fjournals.plos.org%252Fplosone%252Fs%252Feditorial-and-peer-review-process%2523loc-peer-review-history%2F1%2F010f01933d937f8d-58e7117c-50b5-4d8a-a0aa-274a8bf2e47d-000000%2F9ZLdgD6JEnIbuSMrKoY3f1e2qu8v5KJOitJk8cEp7LY%3D185&data=05%7C02%7C%7Cef742d632d6d482b2a3108dd0903fc93%7C2e9f06b016694589878910a06934dc61%7C0%7C0%7C638676634923437108%7CUnknown%7CTWFpbGZsb3d8eyJFbXB0eU1hcGkiOnRydWUsIlYiOiIwLjAuMDAwMCIsIlAiOiJXaW4zMiIsIkFOIjoiTWFpbCIsIldUIjoyfQ%3D%3D%7C0%7C%7C%7C&sdata=rYDznOoiDm0EYr5Nua37v%2BgHNLa0BoHt1pp0%2Fl4Alyw%3D&reserved=0)). If published, this will include your full peer review and any attached files.


If you choose “no”, your identity will remain anonymous but your review may still be made public.


**Do you want your identity to be public for this peer review?** For information about this choice, including consent withdrawal, please see our [Privacy Policy](https://eur02.safelinks.protection.outlook.com/?url=https%3A%2F%2Ftrack.editorialmanager.com%2FCL0%2Fhttps%3A%252F%252Fwww.plos.org%252Fprivacy-policy%2F1%2F010f01933d937f8d-58e7117c-50b5-4d8a-a0aa-274a8bf2e47d-000000%2FCB_F5LG5jfhBbboHkxJHlZtKK3LlO6ts6dMmAoMFjwo%3D185&data=05%7C02%7C%7Cef742d632d6d482b2a3108dd0903fc93%7C2e9f06b016694589878910a06934dc61%7C0%7C0%7C638676634923450788%7CUnknown%7CTWFpbGZsb3d8eyJFbXB0eU1hcGkiOnRydWUsIlYiOiIwLjAuMDAwMCIsIlAiOiJXaW4zMiIsIkFOIjoiTWFpbCIsIldUIjoyfQ%3D%3D%7C0%7C%7C%7C&sdata=qlWGwq2%2BJ4WRbNbnNBh4wUDVocghXn0RLimGCNBMF94%3D&reserved=0).

Reviewer #2: No

Reviewer #4: No

**Response to Reviewer 4’s minor concerns that have been highlighted in comments on the manuscript text.** (Line numbers linked to the comment refer to the reviewer’s annotated manuscript, line numbers in the italic responses refer to the ‘Revised Manuscript with Track Changes’)

**We note that reviewer 4 has read and commented on an earlier version of the manuscript and not the one that was uploaded in March 2024. However, many their comments apply equally to the March 2024 revised manuscript, but a few have already been addressed in the March revision. These are noted in the response below.**

Commented [H1]: What informed the year selection? Why did you limit the search to 2000-2022? (line 34)

*R1 – The scoping review focused on existing practice (line 30), hence a time window of the previous 20 years was set at the start of the project in 2019. This was extended to April 2022 due to delays in submission and review, hence the 23 year window. As this is the abstract it would be inappropriate to go into such a lengthy explanation, suffice it to say that we are focussed on existing practice and have added recent and to line 33 to emphasise the currency of the evidence.*

Commented [H2]: What do you mean by “physical” Please specify some of the physical outcomes (line 44)

*R2 – the words ‘especially in the areas of movement, communication and memory’ have been added to the sentence (line 45).*

Commented [H3]: This sentence needs revision as it appears ambiguous (line 58)

*R3 – sentence amended to clearly link the two halves to remove the ambiguity (line 61).*

Commented [H4]: It will be useful to provide more detailed evidence of the distinction(s) in stroke experience and rehab outcomes between young and elderly stroke survivors (line 61)

*R4 – sentence amended to identify age specific issues around family, relationships, career or employment (lines 63-64).*

Commented [H5]: This sentence is ambiguous (line 62)

*R5 – sentence reworded for clarity (lines 65-67).*

Commented [H6]: The team comprising of who, who and who? (line 139)

*R6 – Team identified by initials (line 150).*

Commented [H7]: Does this refer to the review or previous/ongoing study? (line151)

*R7 – study replaced by YARNS project to clarify (line 165)*

Commented [H8] [H9R8]: What is the nature of the feedback? Kindly describe (line 163)

*R8/9 – sentence modified with addition of ‘critical comment related to their experiences’ (lines 167-168).*

Commented [H10]: 85 what? You mean studies? How many participants were involved and what were the designs of the included studies (line 163)

*R10 – Sentence modified to add ‘articles’ (line 175). Numbers of participants and study designs are given in Table 2 following line179.*

Commented [H11]: I would like to know how many studies that contributed to this and the frequency of each of the physical impact/consequences of stroke among young adults (line 192)

*R11- The studies contributing to this are identified in text by their citation number in this paragraph (lines 193-201). Further analysis is given in Table 4 following line 320 where this applies to rehabilitation programmes. I also note that this is a scoping review providing a broad descriptive picture of the area, we have not undertaken a formal meta-analysis to combine data from the individual papers, which would have been beyond the scope of this review. Further explanation is given in the limitations section (lines 655-672).*

Commented [H12]: I would like to know how many studies that contributed to this and the frequency of each of the social impact/consequences of stroke among young adults (line 212)

*R12 - The studies contributing to this are identified in text by their citation number in this section (lines 228 -279). Further analysis is given in Table 4 following line 320 where this applies to rehabilitation programmes. I also note that this is a scoping review providing a broad descriptive picture of the area, we have not undertaken a formal meta-analysis to combine data from the individual papers, which would have been beyond the scope of this review. . Further explanation is given in the limitations section (lines 655-672).*

Commented [H13]: I would like to know how many studies that contributed to this and the frequency of each of the psychological impact/consequences of stroke among young adults (line 267)

*R13 - The studies contributing to this are identified in text by their citation number in this section (lines 267 -285). Further analysis is given in Table 4 following line 320 where this applies to rehabilitation programmes. I also note that this is a scoping review providing a broad descriptive picture of the area, we have not undertaken a formal meta-analysis to combine data from the individual papers, which would have been beyond the scope of this review. Further explanation is given in the limitations section (lines 655-672).*

Commented [H14]: How was this ascertained? How many studies contributed to this? (line 346)

*R14 – Text modified and a reference added to this sentence (lines366-368). The studies contributing to specific points in this section are identified in text by their citation number (lines 368 -397).*

Commented [H15]: What is the average frequency? (line 381)

*R15 – The papers supporting this statement are identified on line 402. As this is a scoping review providing a broad descriptive picture of the area, we have not undertaken a formal meta-analysis to combine data from the individual papers, which would have been beyond the scope of this review. Further explanation is given in the limitations section (lines 655-672).*

Commented [H16]: What is the frequency? Ow many studies contributed to it? (line 396)

*R16 – The papers supporting this statement are identified in relation to the specific points outlined (lines 416-426). As this is a scoping review providing a broad descriptive picture of the area, we have not undertaken a formal meta-analysis to combine data from the individual papers, which would have been beyond the scope of this review. Further explanation is given in the limitations section (lines 655-672).*

Commented [H17]: How many of them felt this way? How many studies were involved? (line 424)

*R17 – It would appear that this section is from an earlier manuscript and was revised with more detail in the version submitted in March 2024. Please see the section (lines 439-456) for a more extensively referenced and revised discussion of these points. I would re-iterate that we are focussed on a broad descriptive scoping of the area and due to the heterogeneity of the literature it is not possible to undertake a quantitative metanalysis of these points which is acknowledged in the limitations section (lines 655-672).*

Commented [H18]: Conclusion should address the three objectives. To what extent these needs are unmet is not provided (line 627)

*R18 – It would also appear that this section, the discussion, the section on limitations and conclusion is from an earlier manuscript, these have been revised with more detail and a further section on recommendations added in the version submitted in March 2024. Please see these sections from the revised discussion, line 551, to the end of the conclusion (lines 676) for a more extensive critique of the points related to the initial research questions. I would re-iterate that we are focussed on a broad descriptive scoping of the area and due to the heterogeneity of the literature it was not possible to undertake a quantitative metanalysis.*
